# The Bioinspired Prosumer—Interactions between Bioinspired Design Methods in the Prosumer Scope

**DOI:** 10.3390/biomimetics9090539

**Published:** 2024-09-06

**Authors:** Ignacio López-Forniés, Laura Asión-Suñer, Alba Sarvisé-Biec

**Affiliations:** Design and Manufacturing Engineering Department, Zaragoza University, 50009 Zaragoza, Spain; lauraasion@unizar.es (L.A.-S.); albasarvise@gmail.com (A.S.-B.)

**Keywords:** bioinspired design, design methods interaction, prosumer, maker movement, prototyping, domestic fabrication

## Abstract

The emergence of prosumers, who actively participate in designing and producing goods, has generated a growing interest in homemade products. Factors such as design methods, component reuse, or digital fabrication empower prosumer designers to realize their ideas. Although there are cases of bioinspired products manufactured by prosumers, the interactions between bioinspired design methods in the prosumer field have not been addressed from an academic point of view. This article presents a case that combines bioinspired design methods with prosumer characteristics from the perspective of a designer who uses biological research results whilst acting as a prosumer. The proposal is to see whether working on a small scale, without the need for biomimetics experts, and independently, as a prosumer, is feasible and valuable. As a result, a bicycle flashlight is designed with a microgenerator bioinspired by the geometry of samara seeds, and is tested in a wind tunnel. This case shows that the integration of a bioinspired design in prosumer contexts poses unique challenges and requires a multidisciplinary approach. Furthermore, the application of a bioinspired approach in this case has not only provided a certain level of novelty to the final product, but has also improved its efficiency and reduced its financial expenditure.

## 1. Introduction

Bioinspiration uses biological phenomena to stimulate research in non-biological sciences and technology [1]. In particular, bioinspired designs use analogy-making in design, allowing one to identify useful functions or patterns in nature and utilize them in the design of products [2]. The application of bioinspired designs by non-biological experts, whether these are designers or not, expands the design space with new methods and processes looking at nature, since these can be amateurs in the construction of objects but have a special sensitivity towards nature and the environment. Through the use of freely available knowledge, tools, and methods, the interdisciplinarity of the bioinspired process is enhanced, as individuals from diverse disciplines will access these tools and share their own knowledge and resources, which optimizes the process by involving a variety of experts [3].

Prosumers are understood as those users who simultaneously fulfill the profile of producer and consumer. Consequently, prosumer homemade products are those that are designed, developed, and manufactured by the individual consumer usually for their own consumption [4]. The prosumer scope therefore includes profiles such as makers and trends such as DIY, but also users who simply adapt products to satisfy their needs and wants. This vision blurs the traditional boundaries between consumers and producers, allowing end-users to take control of the creative process [5]. It is worth highlighting the lack of design methods by and for the prosumer, where intuition and trial and error prevail. Although there are a large number of tools such as digital fabrication, do-it-yourself, or learning activities [6], the integration of novel methodologies that empower prosumers to apply them in their projects is imperative, alongside the critical task of assessing the ultimate outcomes [7].

Tailored designs address unmet requirements, serving as a means to surmount existing limitations by incorporating essential features or functionalities. Occasionally, prosumer products introduce novelty through their design, even if they do not ultimately penetrate the market [8]. The prosumer and maker movements recycle products by separating their components and materials as a means of sustainability and economic savings [9], integrating aspects of sustainability and circular economy, whilst increasing attachment to the product.

Domestic fabrication serves as a crucial tool enabling prosumers to design objects, advancing them to the prototyping stage with a robust level of development [10]. These objects serve as proof of concept for design validation, fine-tuning functionality, and addressing manufacturing and maintenance aspects [11]. Consequently, this approach enhances product refinement by incorporating details that iteratively improve and update the object throughout its lifespan. This level of control empowers prosumers to manage the entire production process. For designers, it facilitates rapid and streamlined design refinements, independently of external manufacturers.

Design research explores innovative alternatives applicable to design processes, techniques, and models. By integrating various methods, the model used by prosumers is enhanced. Insights from biological systems have uncovered evolutionary strategies that optimize resource efficiency [12,13]. These principles find relevance in prosumer product design, aiming to minimize the environmental impact while maximizing utility. Key aspects of bioinspired design, such as functionality, structures, materials, systems, and forms, can be tailored to align with prosumer characteristics like personalization, user engagement, and sustainability. This customization leads to effective and viable solutions [14].

In many cases, industrial designers lack detailed knowledge of bioinspired design methods, which prevents them from utilizing or even experimenting with these techniques, and flexibility in the mixed methods is a way to improve their use [15]. Prosumers are typically self-taught and do not use structured design methods [16]. Based on these two premises, it may be useful to define a design method that incorporates prosumer behavior, providing an opportunity to combine self-taught creativity [17] and advanced bioinspired design techniques to create an innovative and efficient product [7].

The objective of this article is to demonstrate an effective design approach that facilitates learning through interaction with various methods, leveraging their strengths in the most suitable project phases, proposing the deliberate articulation of interdisciplinary epistemological perspectives and fostering research to develop novel approaches for bioinspired innovation [18]. Additionally, it highlights how novice designers can personalize the process to address the unique requirements of individual users and the specific products they intend to create. Employing a methodology that integrates bioinspired design research, prototyping techniques, and domestic manufacturing, the study outlines a comprehensive design, with development and production processes for prosumer products. As a result, a new community that does not use bioinspired design methods is reached, the bioinspired design philosophy is disseminated, and the prosumer community is provided with access to tools and methods.

The combination of bioinspired design methods in the prosumer scope is presented as a methodological alternative in transforming the economic model, supporting systemic sustainability and the generation of proprietary technologies [19]. The work complies with the Sustainable Development Goals SDO 07 Affordable and Clean Energy, SDO 09 Industry Innovation and Infrastructure, SDO 11 Sustainable Cities and Communities, and SDO 13 Climate Action, by presenting a bicycle flashlight that transforms the mechanical energy produced by wind into electrical energy (SDO 07 and SDO 13), as well as slowing down ephemeral consumption through the promotion of a method based on repair and domestic manufacture that extends the lifecycle of the products (SDO 09 and SDO 11).

## 2. Materials and Methods

This section outlines the proposed design process, where a novice designer adopts the role of a prosumer and utilizes biological knowledge to make the process bioinspired. Each part of the process is described, explaining the rationale behind each decision.

The biomimetic prosumer design process followed is similar to classical product design and development processes, taking as a reference the linear models with iterations between phases [20]. Alternative methods are added, such as bioinspired design [3,21,22], sustainability, and circular economy tools and strategies [23], and, finally, methods related to the prosumer process are added, such as laboratory experimentation and validation practices through prototypes [11,24], and fine-tuning by the optimization of prototypes and completion as finished products [25]. The entire design process is conducted from a consumer standpoint, since the designer will be the one who builds and enjoys the final product. 

**Figure 1:**
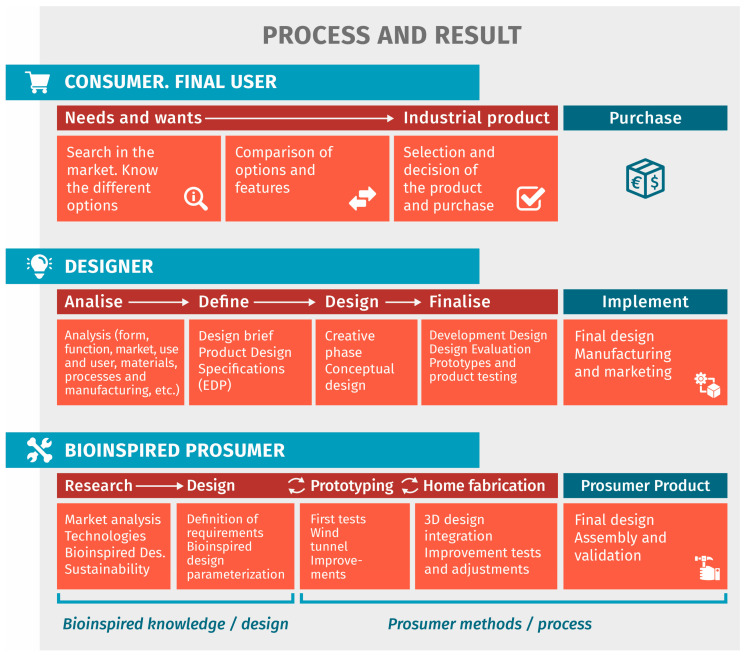
Visions of the design process according to each perspective [3,11,21,22,24,25,26,27].

Figure 1 illustrates the roles that the bioinspired prosumer designer must adopt in the proposed process. The designer acts as a user/consumer, an industrial designer, and a prosumer utilizing bioinspired design resources. The consumer recognizes a need or want, explores market options, and, once informed, starts making decisions based on their initial requirements. After locating the preferred product, they complete the purchase and derive satisfaction from it. From an industrial standpoint, a comparison can be drawn with Bobbe’s research [20], which summarizes diverse design processes into five phases: (1) Research—the project begins with an analysis of consumer needs. (2) Design definition—design requirements are specified, and the concepts are developed. (3) Design—design work and concept refinement take place. (4) Finalization and manufacturing—detailed design is completed, including prototype validation. (5) Implementation—engage in industrial production and subsequent marketing. As for the bioinspired prosumer, the structure of the five phases of the design processes is maintained. However, differences are established due to the main objective, which changes from designing an industrial product to designing a personal product through their own means. Thus, although the first two phases of research and design may be similar to those of a designer, with the notable difference that it is performed with bioinspired knowledge, the process changes significantly in the next three phases. In prototyping, home fabrication, and obtaining final product, the industrial process is replaced by the prosumer process based on constant product improvement.

Prosumer designers can choose from a variety of processes encompassing product design, manufacture, and assembly [26,27]. The prosumer designer, having a primarily self-taught profile, does not seek deep knowledge. Their objective is to understand the concepts related to the knowledge they will apply and to know how to use the specific data required for their design. For this reason, this type of user does not use traditional design methods. Their objective is to solve a problem, and when they lack the specific knowledge to do so, they inform themselves about the necessary subject matter—in this case, bioinspired design. Therefore, the prosumer designer does not employ a specific bioinspired design method. Instead, they utilize the search for bioinspired knowledge to inform their own design, without having to engage in an extensive exploration of the field. This approach allows for the acquisition of only the relevant data necessary for the design, prototyping, and improvement of the product.

Research involves analyzing the prosumer’s own needs and defining the product to be built. At this point, the design space is determined in accordance with bioinspired design and home fabrication requirements. Customized design is the phase in which the initial research findings are obtained, the necessary parameters and dimensions for construction are determined, preliminary tests are conducted, and improvements are implemented. During the home fabrication phase, digital manufacturing techniques are employed, and the ultimate blueprint is constructed by combining all of the elements that will culminate in the final design and production of the prosumer product [28]. Among the design, prototyping, and home manufacturing phases, iterations occur to refine the design based on experimentation and adjustments to the initial requirements.

Table 1 shows the detailed design process carried out from the prosumer designer’s point of view. The selection of a bicycle flashlight was due to the low power generated by a microturbine and the limited air flow speed. The bioinspired flashlight incorporates a small wind generator inspired by the rotating seeds of the samara. The prototypes and final design underwent laboratory validation through wind tunnel testing and the bioinspired flashlight was subsequently constructed via rapid prototyping. The electrical circuit was meticulously engineered to ensure optimal performance and compliance with relevant regulations. Furthermore, the design process embraced a circular economy and sustainability principles, including component recycling, reuse, life extension, and repairability.

### 2.1. Research

Initially, customers, creators, and prosumers must recognize the resources available on the market, e.g., existing solutions, technology, materials, and implemented manufacturing methods. This research focuses on flashlights, specifically a bicycle flashlight that transforms the mechanical energy produced by wind and domestic wind turbines into electrical energy.

Websites dedicated to makers [29] compile projects and display the results, some of which are classified by project and others offer knowledge and experience. These websites and Web of Science (WoS) have been searched with the search terms ((“bicycle” OR “bike”) AND (“flashlight” OR “head light” OR “torch” OR “lantern”)).

Regarding the design process, a search in the WoS database is performed with the terms ((“bioinspired” OR “biomimetic” OR “biomimicry”) AND (“design method” OR “design model”)) to determine design models that apply a bioinspired design. Also searched with the terms ((“bioinspired design” OR “biomimetic” OR “biomimicry”) AND ((prosumer) OR (maker movement) OR (maker community)) to determine what work has been performed in the prosumer field with inspiration from nature. Additionally, papers about “seed dissemination strategies”, “rotary seeds”, and “seed flight” provide knowledge of nature strategies for a bioinspired design concept created by the prosumer.

A literature review of microturbines is carried out, with web searches for micro-wind turbines, to analyze their dimensions as well as their functional and technical characteristics. Also, a WoS search is carried out with the terms ((“micro” OR “home” OR “domestic”) AND (“wind turbine”) AND (review)).

With regard to the study of sustainability, methods that are easy to apply for a prosumer designer are sought, such as the principles of circular economy [23,24]. Examples and cases such as the separation and recovery of components, and the design for the interchangeability of components, testing, and bioinspiration are sought [25].

### 2.2. Customized Design

The preliminary design of the blades by a detailed analysis of efficient natural structures, exemplified by the different flying seeds, is carried out [26]. We engage in parameterization and three-dimensional modeling based on initial outcomes. Through iterations, we emulate aerodynamic patterns and shapes within these structures [26,27], optimizing the shape and angle of the blades, as well as the air force acting on the propeller.

### 2.3. Prototyping

The initial tests involve manual construction and 3D printing, allowing for adjustments to validate the blade shape, size, and functionality. Subsequently, experiments occur within a controlled environment, utilizing an open-circuit wind tunnel. These tests assess the design performance and power generation capabilities across varying airflow speeds.

Regarding the electrical circuit, an initial design can be formulated by measuring the energy efficiency. Various electrical load combinations, including LEDs and resistors, are tested. The validation process encompasses individual component testing and an assessment of their interactions within the entire system. The collected data serve as a robust foundation for optimizing and refining blade design and electrical circuitry (complete data in Tables S1–S3).

### 2.4. Manufacturing

The final geometric design entails a visual representation and modeling of the component shapes and dimensions. Using computer-aided design (CAD) tools, SolidWorks 2022, we precisely integrate the validated prototypes into detailed, accurately dimensioned 3D models. Accurate geometry is paramount to ensure proper component fit and efficient functionality. Employing advanced manufacturing techniques, these create housings and enclosures that not only meet esthetic criteria but also provide essential protection and functionality.

### 2.5. Validation

The assembly, fitting, and testing phase constitutes the culmination of the construction process. During this stage, all components are assembled, mounted onto the bicycle, and fine-tuned to ensure seamless operation. Tests are then conducted under real-world conditions to assess the prototype’s performance, energy efficiency, and alignment with the stated objectives. This comprehensive testing validates both the functional operation and practical feasibility of the product.

## 3. Results

We summarize in this section the most important results of each design phase for the proposed design, prototype, and manufacture of an autonomous and rechargeable energy flashlight for bicycles.

### 3.1. Research

Market research, design, scientific literature review, and the creation of a design brief are not common practices among members of the maker community, who typically turn to community websites to see examples and cases similar to their project of interest. An example of this is the website Instructables [30], which features over 100 examples of homemade turbines manufactured with recycled materials or digital fabrication. However, despite the high number of results, none of them are of bioinspired design.

#### 3.1.1. The Bicycle Flashlight Market

Forty-eight results were obtained in WoS and, once filtered, five of them were useful. One refers to a patent study of LED flashlights for bicycles and indicates that there are patents for microturbines as a power source [31]. The other four results serve for the definition of the design requirements and they refer to the optics used, the type of emitter used, and to a flashlight designed for a 1-W power LED. Given the existence of related patents, a search was made in WoS and Google Patents with forty-two results, but only 4 related patents were found. There is similarity in terms of the design and functionality but no examples of commercialization have been found.

**Figure 2:**
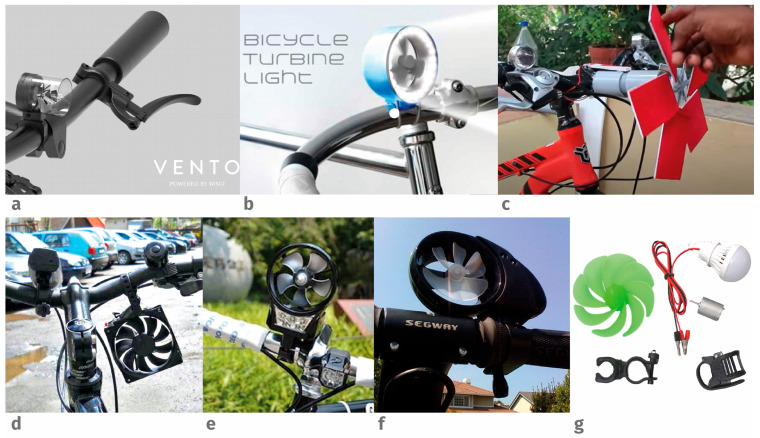
Analysis of bicycle flashlights on the market. Conceptual designs (**a**,**b**); Vento Bioinspired design (**a**); Prosumer Designs (**c**,**d**); Manufactured and marketed (**e**,**f**); Kit for DIY model (**g**).

During the market research process, we select from among the various examples identified those that are similar to the product under design (Figure 2), but some have not yet reached the prototyping or commercial phases. The selection was made to obtain examples that encompassed aspects of biomimetics, conceptualization, and prosumer design and manufacturing. The first example, called Vento, has a bioinspired working principle and incorporates aspects of energy sustainability (see Figure 2a). The other is only a sketch and 3D representation of a pinwheel, as shown in Figure 2b. The windmill generator cycle is an example of prosumer manufacturing using recycled materials to build the propeller and the chassis (Figure 2c). Another example, shown in Figure 2d, uses a computer fan to power a flashlight in a very artisanal way.

In Japan, the flashlight marketed by Thanko (Figure 2e) starts generating energy at 15 km/h. In the United States, the HYmini personal wind turbine is a portable power supply that can be topped up with wind energy (Figure 2f). Finally, the Mini Wind Generator Wind Turbine and Portable Phone Charger, is a mountable kit that can adapt (Figure 2g).

We have selected seven examples from the obtained results based on three criteria: whether the product is conceptual or already produced; whether it has bioinspired characteristics; and whether its production is domestic (Figure 2). Two concepts did not reach the prototyping phase (a and b), one of which was bioinspired (a). Two examples with artisanal construction coincided with the concept of prosumer design (c and d). Two examples were marketed and have a series of characteristics similar to the requirements set by the brief (e and f). And, there was a kit that could be copied and created by a prosumer designer from recycled parts (g).

#### 3.1.2. Understanding Microturbines Operation and Performance

Numerous micro-wind turbine variants exist, and abundant online and scientific resources facilitate an understanding of the factors influencing their design [32,33]. Noteworthy projects encompass both horizontal turbines employing computer fans and vertical turbines featuring curved or cup-shaped blades. Additionally, tutorials elucidate diverse electrical circuits for energy generation and storage. From an environmental perspective, wind turbines yield energy without pollution. Their performance hinges on their blade geometry, quantity, diameter, angle of attack, generator resistance, and Reynolds number [34].

Microturbines with a smaller diameters increase their torque, thereby maintaining revolutions and increasing power. However, it is essential to ensure a minimum torque to overcome the initial friction of the generator, even at low speeds, to prevent the load from stopping the rotor’s rotation [32].

According to the classification of horizontal axis wind turbines (HAWTs) [33], micro-scale turbines can reach about 0.250–1.4 kW with diameters ranging from 0.5 to 1.2 m, although they are dedicated to electricity production for domestic consumption. Microturbines dedicated to bicycles require only 1 to 3 watts, allowing for a drastic reduction in diameters and swept area.

The theoretical power *P* that a HAWT can generate is calculated using Formula (1), where *r* is the rotor radius (m) and *V* is the wind speed (km/h). And, the power coefficient *C_p_* (Formula (2)) is the generator power relative to the theoretical power, where *P_t_* is the real power generated by the turbine, the product of the voltage, and the current produced. For micro-wind turbines, power coefficients are about 0.25 or greater compared to large turbines, which have values of around 0.45 [35]. Once the data from the wind tunnel generation tests are obtained, the performance and power coefficients can be compared.

*P* = 0.04143 × *r*2 × *V*3 (1)

*C_p_* = *P_t_*/*P* (2)

Despite the existence of multiple methods for designing horizontal axis wind turbine (HAWT) blades, most rely on mathematical models and finite element analysis. However, there is no single methodology documented in the scientific literature; rather, each researcher adopts the design methods they are most familiar with [36].

#### 3.1.3. Bioinspired Design

In the realm of industrial design, there is a burgeoning interest in adopting a bioinspired approach [37,38,39,40], leveraging principles and strategies from nature to foster innovation and sustainable solutions [12]. However, its specific application within the prosumer domain remains unestablished. Biological analogies are commonly selected, since these significantly increase the novelty of designs compared to other analogies but there is no significant difference between biological analogies and those spanning different domains [41].

The WoS search yielded 135 results (see Section 2.1) which, once filtered, found one review article on bioinspired design methods and six articles explaining the different types of design models. These results show two approaches: the first, starting from a problem and solving it thanks to inspiration from nature; and the second, using a solution in nature to apply it to the design of a new product [13]. Only one article was found that connects bioinspired design with the maker movement through a STEAM (Science, Technology, Engineering, Arts and Mathematics) program for the professionalization of students [42], which focuses on the bioinspired design process for the materialization of ideas through the prototyping and construction of physical models.

Regarding the seed dissemination strategies, samara seeds are an example used by certain trees to send seeds far from their origin [43] since they are designed to glide efficiently through the air using wind energy. There is proven information on their geometry and weight that allows the design of turbine blades. They are composed of wing and seed parts, presenting the ideal characteristics for displacement: low descent speed, high turning speed, and low pitch angle [44,45]. There are samaras with one or two seeds and with one or more wings. Those with more than one wing glide with a slower descent rate but sometimes do not rotate [46]. The flight behavior of samaras can be analyzed in a vertical wind tunnel through tests using both real samaras and simulated models, determining the autorotation and descent speed that depend on the location of the center of gravity, as influenced by various geometric and loading factors [45].

**Figure 3:**
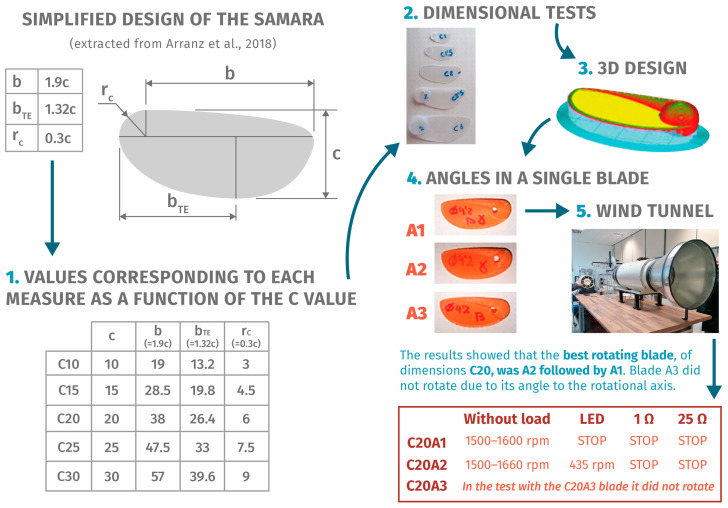
The prototyping and testing process for propellers based on samara blades [34].

Based on the information found, the search for information was focused on maple seeds. Its geometry consists of an elongated, thin wing with a reinforced edge, which at one end has the heaviest part where the seed is located [34,47,48]. All measurements are based in relation to the width of the seed (value *c* in Figure 3). It is assumed that the seed has a constant density and that the wing is completely flat. Its thickness is also related to the measurement *c*. The accessibility and cost-effectiveness of 3D printing technology enable studies and facilitate experimental testing. Previous research has employed artificial samaras [48], but utilizing 3D printing to replicate seeds and study their aerodynamics can expedite these investigations.

The study by Zakaria [43] compares the data for the same type of seeds with shape variations and establishes the importance between the weight of the seed and its aerodynamic properties, although it raises doubts about their application in a 3D-printed blade due to the change in density. Several studies applied this type of design to drone blades to test their efficiency by varying the radius and pitch angle with respect to the axis of rotation [47] and optimizing stability in constant wind for three-bladed rotors [49].

Some cases of rotating samaras with more than one wing have been found [50], suggesting the potential for bioinspired rotors with 2, 3, or 5 blades, considering their dependence on the center of gravity.

One case was found to be inspired by bird wings to achieve greater robustness, although in this instance, flexible wings with a profile adaptable to movement are necessary. This approach offers intriguing elements but proves challenging to implement in prosumer design, particularly for novice designers [51]. In pursuit of flexible materials and adaptable geometries, an example involves textures and flexible blades. These modifications include the corrugated blades inspired by the wing structure of dragonflies, and flexible blades inspired by the wing adaptations of birds and insects to varying air conditions [52].

The utility of small wind turbines has spurred research into bioinspired blade designs aiming to explore unconventional solutions [53], such as the three-bladed rotor inspired by the seed of a tree known as Triplaris Americana [54], or adaptations of blades featuring tubercles inspired by the humpback whale [55].

Regarding theoretical yields, the power coefficient *C_p_* of the maple seed was observed to be 0.59, comparable to the range of 0.45–0.48 for many wind turbines and close to the Betz limit of 0.593 [56].

#### 3.1.4. Design Brief

The pillars of the design for the bioinspired bicycle flashlight are microturbines, which are natural analogies for blade design and digital fabrication. Therefore, the prosumer designer needs to satisfy the requirements for knowledge and data utilization. Prosumer design briefs define their own needs and wants as a final user as well as the product design specifications (Table 2). In this type of project, the brief can be defined as self-commissioned [17].

### 3.2. Custom-Made Design

Figure 3 presents the dimensions for the construction and tests of the first blades and the artificial seeds made of plastic sheets. The study by Arranz [34] provides the data for building a replica of the seed, and the tests carried out confirm that it is necessary to design a 3D replica of the seed in various dimensions. Finding the center of gravity in manual construction can be challenging, and several characteristics depend on its accurate determination [45]. For the first tests, we looked for old toy motors, computer fans, hair dryers, etc.; a flashlight for bicycles and spotlights to take out the LED and convergent lenses; rechargeable batteries and battery holders or cables to connect the set.

Based on the model of Arranz [34], a modification of Zakaria’s study is included [43]. At the upper edge, a rib is included to reinforce its structure and balance the center of masses. For the thickness, the measure given by the value of c was not adopted because it was too thin and to avoid problems of durability. The shape of the seed simulation was modeled with SolidWorks 2022 educational version (Table 3, complete data in Table S4) and the center of mass was used as the axis reference.

Once the 3D propeller model is fully defined, the angles of inclination must be included. In Zakaria’s study [43], the angles are provided with respect to the vertical axis passing through the center of mass of the seed, pitch angle (θ = 24′77°, horizontal plane), and draft angle (β = 15′72°, vertical plane). Three different types of blades were designed to determine whether the angle affects the rotor speed, the output voltage, and the electrical power (Figure 3): model A1 (angles θ and β), model A2 (only angle θ), and model A3 (only angle β).

### 3.3. Prototyping

The model was 3D-printed for subsequent testing. Tests were conducted using a homemade open-type wind tunnel with 10 speed settings ranging from 1 m/s to 13.33 m/s, generating a linear airflow towards a movable support. As the maximum speed of a bicycle in the city is 30 km/h [57], the seventh (35.3 km/h, 8.33 m/s) and tenth (48.2 km/h, 13.33 m/s) positions will be used for the tests to evaluate the maximum flow.

#### Conceptual Design Review

After the initial tests, it was concluded that the blade with only the θ angle of 24.77° in model C20A2 performed the best. Previous experiments with the design of the experimental horizontal axis wind turbines utilized smaller angles of 15°, 18°, and 20°, yielding better power coefficients [33]. The angle β does not generate enough torque to start the rotor’s rotation and is therefore discarded. The recycled motor is not able to generate enough power to light the 1 W LED.

Changes are proposed for the following tests, such as testing the generator used and other similar generators on a test bench to check the power, current, and voltage they can generate; reducing the impedance of the circuit; increasing the diameter of the blade so that the motor has more torque; and adding more blades, creating a set of propellers, going from one and two blades to three and five blades.

### 3.4. Experimentation and Final Validation

**Figure 4:**
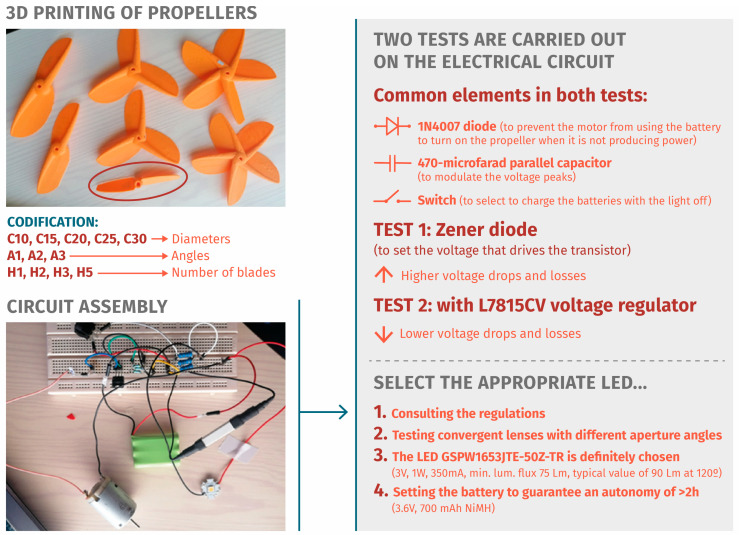
Electrical circuit test for final validation.

For the final experimentation, the C25 model is manufactured with two, three, and five blades and the single angle θ. A universal brushed motor is obtained from a recycled dryer of which only the voltage (36 V) and speed (19,000 rpm) are known. A test is carried out to test the characteristics of the generator by connecting it to a reference motor that will be responsible for moving the rotor. According to results, with a 25 Ω resistor, 1 watt of power is achieved at 3871 rpm, 5.1 V, and 208 mA. Observing the results of the previous tests, this motor with the 3H-C2’5 model reached 3500 rpm and 6.4 V; for this reason, it was decided to carry out more tests in the wind tunnel to verify that the generator is valid. In the last test, the propeller models C25A2H2, C30A2H2 C25A2H3, C30A2H3, C25A2H5, and C30A2H5 are used (see Figure 4). In addition, a drone propeller was printed, from the website cults3D, with a radius of 25 mm.

The optimal outcomes were achieved using the H5C30 rotor coupled with a 5 W LED, where the resistor’s influence on power output was negligible. Additionally, the H2C25 rotor exhibited a superior power coefficient, aligning closely with the findings reported in comparable studies [33] that evaluated larger wind turbines ranging in diameter from 0.5 to 2.2 m, which evaluated larger wind turbines ranging in diameter from 0.5 to 2.2 m. The optimal efficiency range for horizontal axis wind turbines (HAWTs) typically falls between 50% and 60% [33]. However, the performance of the bioinspired rotor in this study falls significantly short of these benchmarks. This disparity underscores the ongoing challenge and potential for further enhancement in bioinspired wind turbine technology.

### 3.5. Manufacturing and Home Fabrication

**Figure 5:**
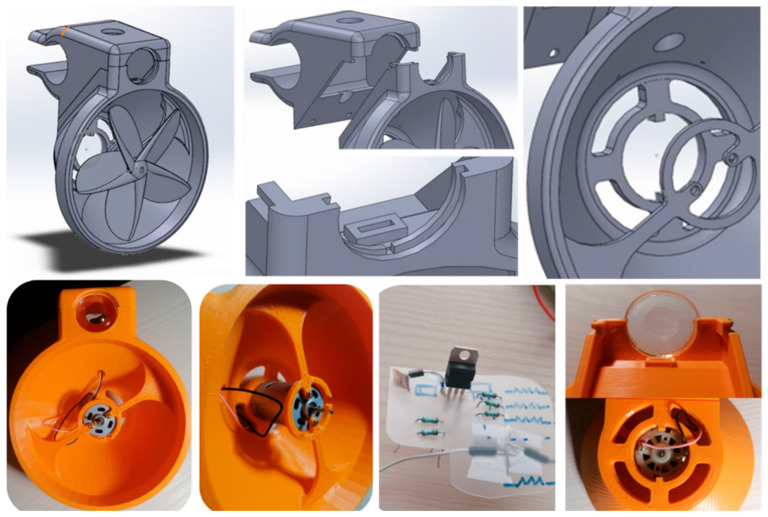
Design and 3D printing of the first complete prototype.

To manufacture the prototype, the same printer used in the propellers is used, and the printer’s specifications and printing parameters can be found in Table S5. As it is necessary to have a space inside in which to place the circuit, it will be divided into two parts that can later be fixed (see Figure 5). With this first complete prototype, an improvement in manufacturing can be seen in the fitting of the parts, the fixing of the generator, the screwed connection, and the fastening to the handlebars. And, an improvement can be observed in the assembly to pass the cables whilst avoiding breakage and pulling.

#### Final Assembly, Adjustment, and Validation

**Figure 6:**
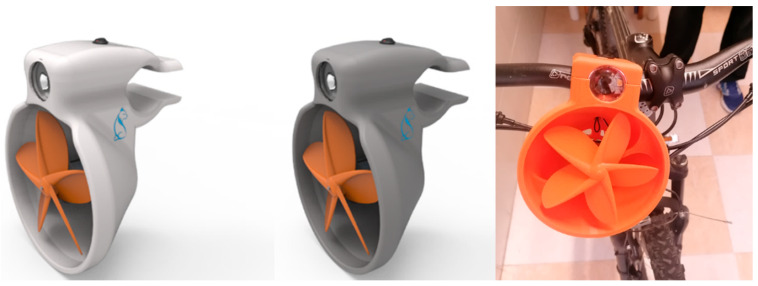
Final 3D design.

The 3D design undergoes updates to incorporate proposed enhancements, resulting in improved esthetics characterized by organic and fluid shapes. Adjustments are made to the anchorages for seamless bicycle mounting, and initial real-world tests are conducted. These tests validate both battery charging and night lighting functionalities. Figure 6 visually presents the final 3D design alongside the fully assembled prototype affixed to the bicycle.

Notably, meticulous attention ensures the absence of assembly or adjustment errors. Multiple prototypes were printed, allowing necessary modifications to accommodate all components. To safeguard the electrical elements from rain or condensation, an airtight enclosure is introduced. Additionally, the LED is isolated using a silicone bead. Enhancing user comfort, an elastomeric material band is incorporated to absorb vibrations and prevent rotational movement.

For future iterations, a specification will be developed to optimize the volume and dimensions, aiming for greater compactness. Furthermore, for potential impact scenarios, such as drops, the generator was strategically placed behind and under the handlebars and reinforced with thicker casings to mitigate damage.

## 4. Discussion and Conclusions

Despite widespread use in various fields, the integration of bioinspired design into prosumer contexts poses unique challenges, necessitating a multidisciplinary approach. This underscores that the differences between a genuine prosumer and an academically trained novice designer primarily stem from their approach and preparation. The prosumer has no access to laboratories, specialists, biologists, etc. The initial lack of knowledge regarding research and interpretation of scientific literature on bioinspired design necessitates additional effort. For this reason, the prosumer has to implement the results of others (specialists or not) with a research phase. He must consider what he can do with what others have done. Other prosumers might opt for a conventional propeller, as seen in the examples found, rather than exploring optimization and improvement through bioinspired design as in other examples [58,59]. In this case, the prosumer experiments by copying (the design of the blades), transforming (adaptation of a rotor with 1, 2, 3, 5 blades, elimination of one of the angles), and combining their manufacturing experience with the results of the experiment, which makes it possible to refine the final product. In this way, the prosumer designer’s familiarity with prototyping and validation tests simplifies the laboratory experimentation.

In the context of a bicycle flashlight, a novel product concept emerges with a degree of innovation. Harnessing wind to generate electrical energy, rather than relying on conventional dynamos, not only reduces the cyclist’s effort but also proves to be a more cost-effective alternative [32]. This approach highlights the efficiency gains and economic advantages associated with wind power utilization over traditional methods. Other prosumer designers can easily replicate and enhance this concept. The primary limitation lies in wind tunnel usage, but alternative tests can be conducted using different instrumentation. Prosumers often rely on trial-and-error techniques, using real-world testing to identify errors and facilitate improvements. Unlike purchased products, prosumer creations remain dynamic and adaptive. Furthermore, prosumer products can continually evolve to meet specific or future needs.

The development of prosumer goods and iterative testing yields knowledge that can be shared among designers and prosumers for design modification, adaptation, and personalization. The designer’s market exploration phase finds robust support from both academia and industry. However, a critical question arises: Do prosumers need guidance or assistance during the design research phase to fully grasp their needs and explore viable alternatives before embarking on the design process?

In general, academically trained designers have a wealth of options when they draw from diverse design methodologies, selecting the most effective elements from each. For prosumers who embrace trial-and-error approaches, integrating novel design methods becomes an intriguing learning journey. Yet, this pursuit relies on voluntary exploration, often facilitated through community-based information exchange, which may have its limitations.

Based on the results meeting the initial expectations and satisfying the objectives of lighting, electrical performance, and the application of bioinspired processes, future efforts should focus on adjusting the rotor pitch angle and testing other generators for improved efficiency. Additionally, exploring the design potential of vertically oriented bioinspired turbines, which offer a higher performance, is an intriguing prospect.

## Figures and Tables

**Table 1:** Design process for the bioinspired prosumer.

Phases	Objectives	Actions and Methods
Research	Understanding the market for flashlights and microturbines Search for bioinspired references; know the biological principle of the seeds Understanding sustainability and circular economy issues Defining needs	Market analysis, similar features/functionality Microturbine analysis Bioinspired case studies Sustainable design rule integration Writing the brief
Customized Design	Conceptual design; custom-made design Establish the dimensions of the blades for the first tests Know how the wind tunnel works	Conceptual design; preliminary design of blades and schematic or grid of component blocks Recover old motors, LED, and lenses for testing
Prototyping	Experimentation and validation Tests with recovered generators and various LED final tests Calculation of electrical components	Three-dimensional printing, first tests Wind tunnel laboratory experimentation Electrical circuit design and optimization
Manufacturing	Carrying out the geometric design Integrating all components Three-dimensional printing final esthetic design	Three-dimensional printing; esthetic design Adjustment of anchorages on bicycles
Validation	Actual assembly, adjustment and testing	First real tests of the prosumer product

**Table 2:** Product design main specifications for the bioinspired bicycle flashlight.

Component	Specifications
Light	One 1–3 W LED; focusing/converging lens
Legal	A white light source that must reach at least 150 m with an illumination of 4–60 candelas (12.6 Lm to 188.5 Lm at 120°) and facing forward in the direction of the axis of motion [57]
Power	Autonomous charging by bicycle movement; micro-wind turbine; use of rechargeable batteries type AA or AAA
Circuit	Electronic circuit board for battery charging and illumination; selector for charging or charging with illumination
Generator	Recycled motors from old appliances
Fabrication	Three-dimensional printing; recycled and recyclable materials
Sustainability	Introduce sustainability rules through the recovery and reuse of materials and components

**Table 3:** Data and results in the wind tunnel.

	b = c·1.9 (m)	Swept Area (m^2^)	Theoretical Power *P* (W)	Resistor (Ω)	Voltage (V)	Current (mA)	Speed (r.p.m)	Turbine Power *P_t_* (W)	*C_p_* = *P_t_*/*P*
H5C30 (led 5W)	0.057	0.0102	5.91	25	7.8	160	5762	1.248	0.211
H5C30 (led 5W)	0.057	0.0102	5.91	56	9.4	120	6153	1.128	0.191
H2C25 (led 5W)	0.048	0.0071	4.10	25	7.24	142	5075	1.028	0.250
H2C30 (led 1W)	0.057	0.0102	5.91	25	8.2	120	5870	0.984	0.166
H5C30 (led 1W)	0.057	0.0102	5.91	25	7.7	125	7000	0.963	0.163
Dron (led 5W)	0.025	0.0020	1.14	39	4.87	50	2860–2900	0.2435	0.214

## Data Availability

All relevant data are contained within the paper and supporting materials.

## Supplementary Materials

The following supporting information can be downloaded at: https://www.mdpi.com/article/10.3390/biomimetics9090539/s1, Table S1: Results of the tests carried out according to the power coefficients. Table S2: Results of tests carried out with motors. Table S3: Luminescence test. Table S4: Wind tunnel results. Table S5: Data of the printer used for prototyping.
