# Supplementary material for: The Bioinspired Prosumer—Interactions between Bioinspired Design Methods in the Prosumer Scope
Source: Biomimetics (Basel). 2024 Sep 6;9(9):539. doi: 10.3390/biomimetics9090539 (PMC11429633; doi:10.3390/biomimetics9090539)
Supplement: Supplementary file 1 [file biomimetics-09-00539-s001.zip › biomimetics-3104916-supplementary.pdf]

biomimetics

MDPI

Supplementary Data

The Bioinspired Prosumer—Interactions between Bioinspired Design Methods in the Prosumer Scope

Ignacio López-Forniés *, Laura Asión-Suñer and Alba Sarvisé-Biec

Design and Manufacturing Engineering Department, Zaragoza University, 50009 Zaragoza, Spain; lauraasion@unizar.es (L.A.-S.); albasarvise@gmail.com (A.S.-B.)
* Correspondence: ignlopez@unizar.es; Tel.: +34-617372657

Table S1. Results of the tests carried out according to the power coefficients

	b (m)	Swept area (m2)	Theoretical power (P)	Resistor (Ω)	Voltage (V)	Current (mA)	Speed (r.p.m)	Turbine Power Pt (W)	Cp= Pt/P
H5C30 (led 5W)	0,057	0,0102	5,91	25	7,8	160	5762	1,248	0,211
H5C30 (led 5W)	0,057	0,0102	5,91	56	9,4	120	6153	1,128	0,191
H2C25 (led 5W)	0,048	0,0071	4,10	25	7,24	142	5075	1,028	0,250
H2C30 (led 1W)	0,057	0,0102	5,91	25	8,2	120	5870	0,984	0,166
H5C30 (led 1W)	0,057	0,0102	5,91	25	7,7	125	7000	0,963	0,163
Dron (led 5W)	0,025	0,0020	1,14	39	4,87	50	2860-2900	0,2435	0,214
			35,28						

Table S2. Results of tests carried out with motors

Resistance (Ω)	Voltage (V)	Current (mA)	Motor speed (r.p.m.)	Power (mW)
0 Ω	3	1550	-	-
0 Ω	3,48	1770	-	-
0 Ω	4,55	2330	-	-
0 Ω	5,4	2750	-	-
0 Ω	6,7	3450	-	-
0 Ω	7,5	3880	-	-
0 Ω	8,13	4180	-	-
0 Ω	8,72	4480	-	-
9,75 Ω	2,25	54	1446	121,5
9,75 Ω	3,4	87	2230	295,8
9,75 Ω	5	126	3313	630
9,75 Ω	5,8	147	3840	852,6
25 Ω	1,9	80	1420	152
25 Ω	2,9	120	2150	348
25 Ω	4,3	175	3206	752,5

25 Ω	5,1	208	3871	1060,8
50 Ω	2,3	43	1450	98,9
50 Ω	3,5	69	2230	241,5
50 Ω	5,2	103	3333	535,6
50 Ω	6,1	120	3881	732
100 Ω	2,62	25	1477	65,5
100 Ω	4,87	48	2738	233,76
100 Ω	5,75	57	3273	327,75
100 Ω	6,57	65	3770	427,05
100 Ω	7,2	73	4159	525,6

Table S3. Luminescence test

Angle	Candelas	Lumens	Angle (rad)
90	4	7,361	1,571
60	4	3,367	1,047
45	4	1,913	0,785
30	4	0,856	0,524
90	40	73,612	1,571
60	40	33,671	1,047
45	40	19,131	0,785
30	40	8,564	0,524
90	60	110,418	1,571
60	60	50,507	1,047
45	60	28,697	0,785
30	60	12,846	0,524

Table S4. Wind tunnel results

Resistance (Ω)	Propeller	Tunnel level	Voltage (V)	Current (mA)	Speed (r.p.m.)	Power (mW)	LED
-	Dron	10	7.5-7.35	-	3930-4030	-	LED BLUE 5W
220			6,7	18	3720	120,6	
56			5,28	44	3090	232,32	
39			4,87	50	2860-2900	243,5	
20			4,05	55	2460-2480	222,75	
9.75			3,56	58	2220-2200	206,48	
220	C25A2H2	10	12,05	40	7250	482	LED WHITE 1W
	C30A2H2		11,2	40	6740	448	
	C25A2H3		10,5	40	6770	420	
	C30A2H2		10,9	44	6530	479,6	
	C25A2H5		11,60	60	7014	696	
	C30A2H5		11,1	60	6765	666	
56	C25A2H2	10	8,2	90	5350	738	LED BLU E 5W
	C30A2H2		9,25	105	6076	971,25	
	C25A2H3		9,78	55	6400	537,9	
	C30A2H2		9,6	60	-	576	

	C25A2H5		8,91	100	5950-6000	891	
	C30A2H5		9,4	120	-	1128	
25	C25A2H2	10	7,24	142	5075	1028,08	LED BLUE 5W
	C30A2H2		8,38	70	2854	586,6	
	C25A2H3		7,2	107	5370	770,4	
	C30A2H2		7,94	121	5810	960,74	
	C25A2H5		7,6	131	5851	995,6	
	C30A2H5		7,8	160	5762	1248	
25	C25A2H2	10	6,15	120	7070	738	LED WHITE 1W
	C30A2H2		8,20	120	5870	984	
	C25A2H3		7,08	125	4825	885	
	C30A2H2		7,88	100	5160	788	
	C25A2H5		7,26	130	5300-5500	943,8	
	C30A2H5		7,7	125	7000	962,5	

Table S5. data of the printer used for prototyping

Model	Artillery Sidewinder X1
Technology	FDM (Fused Deposition Modeling)
Construction space	300x300x400 mm
Extruder temperature	240ºC
Filament diameter	1'75 mm
Program	Ultimaker Cura
Construction speed	30 mm/s (First five layers)<br>80 mm/s (Subsequent layers)
High	0,2 mm (First five layers)<br>0,32 mm (Subsequent layers)
Tolerance	+ 0, 32 mm
Material	PLA Ø 1’75 mm
